# Left inferior temporal hemorrhage due to cerebral amyloid angiopathy mimicking semantic dementia

**DOI:** 10.1007/s10072-025-08358-6

**Published:** 2025-07-11

**Authors:** Kazuto Katsuse, Daiki Yashita, Kazuo Kakinuma, Masashi Hamada, Tatsushi Toda, Kyoko Suzuki

**Affiliations:** 1 Department of Neurology, Graduate School of Medicine, The University of Tokyo, 7-3-1 Hongo, Bunkyo-ku, Tokyo 113-8655 Japan; 2 Department of Behavioral Neurology and Cognitive Neuroscience, Tohoku University Graduate School of Medicine, Sendai, Japan; 3 National Center Hospital, National Center of Neurology and Psychiatry, Kodaira, Japan

## Dear editor

Semantic dementia (SD) is a clinical syndrome characterized by progressive loss of semantic memory, including impaired understanding of words, objects, and concepts, due to neurodegenerative pathology [1, 2]. SD is a semantic variant [1] in the context of primary progressive aphasia (PPA) characterized by impaired confrontation naming and single-word comprehension, with relatively preserved speech production and repetition. Beyond linguistic deficits, SD disrupts multimodal semantic cognition, including object decisions and the ability to recall object colors and visual imagery [2]. SD is commonly associated with frontotemporal lobar degeneration (FTLD), particularly TDP-43 type C pathology affecting the anterior temporal lobes. Hallmark features, such as consistent two-way anomia resistant to phonemic cues and impaired nonverbal object knowledge, are rarely observed following focal brain lesions [3]. Here, we report a case of SD-like semantic memory impairment following a subcortical hemorrhage in the left inferior temporal region due to cerebral amyloid angiopathy (CAA). Although serial magnetic resonance imaging (MRI) over 8 months revealed new hemorrhagic lesions corresponding to the onset of semantic deficits, the preexisting anomia and the progressive course of CAA implied gradual decline rather than sudden onset. This case emphasizes the need to consider vascular pathology such as CAA, often coexisting with Alzheimer’s disease (AD), in the differential diagnosis of atypical PPA, and underscores the diagnostic utility of susceptibility-weighted imaging (SWI).

**Fig. 1:**
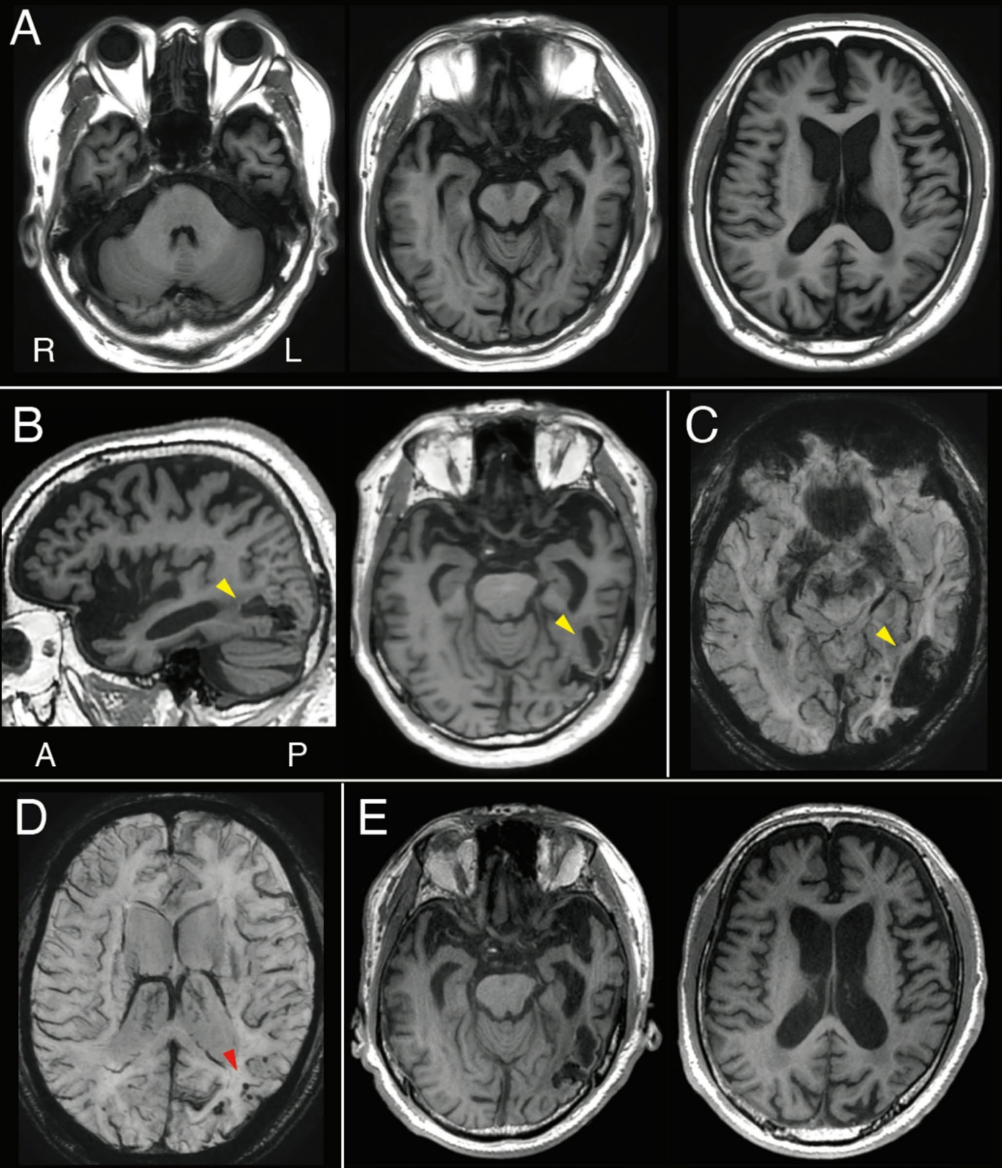
Neuroradiological findings. **A**: Magnetic resonance imaging (MRI) conducted eight months before revealed mild diffuse atrophy without evidence of cerebral hemorrhage. **B**: Follow-up MRI shortly following the initial visit showed subcortical hemorrhage in the left posterior inferior temporal region, which was absent on the earlier MRI (yellow arrow). **C**,** D**: Susceptibility-weighted imaging (SWI) confirmed the hemorrhage in the same region (yellow arrow) and additionally revealed multiple microbleeds, predominantly in the left parietal, occipital, and temporal cortices (red arrow). **E**: Follow-up MRI conducted 20 months following the initial visit showed progressive atrophy in the bilateral medial temporal lobes

An 80-year-old right-handed Japanese man presented with a two-year history of progressive language impairment. Eight months earlier, he underwent brain MRI at a local clinic due to word-finding difficulties, which revealed mild diffuse cerebral atrophy without evidence of hemorrhage (Fig. 1A). Six months before presentation, he abruptly developed reading difficulties and discontinued his hobby of reading. Word-finding difficulties progressed rapidly, and he began asking about the meanings of common words (such as “What is a plastic bag?”). He was referred to the neurology department for further assessment.

Neurological examination revealed fluent aphasia with severe anomia, circumlocution, and semantic paraphasia (Supplementary Table 1). The Japanese version of the Western Aphasia Battery showed impaired comprehension and severe naming deficits unresponsive to phonemic cues, with preserved repetition and motor speech; the Aphasia Quotient was 62.3. The Test of Lexical Processing in Aphasia, a standard assessment for examining Japanese lexical abilities, revealed a poor naming accuracy (30/200) and auditory comprehension (106/200), with consistent two-way anomia. During the task of line-drawing naming, he frequently reported unfamiliarity with the images, saying, “I have never seen anything like this drawing before.” Similarly, during the auditory comprehension task, the patient expressed unfamiliarity with common words (such as “eraser”). Impaired performance on the lexical decision and the proverb completion tasks supported the diagnosis of SD over non-neurodegenerative transcortical sensory aphasia [3].

**Fig. 2:**
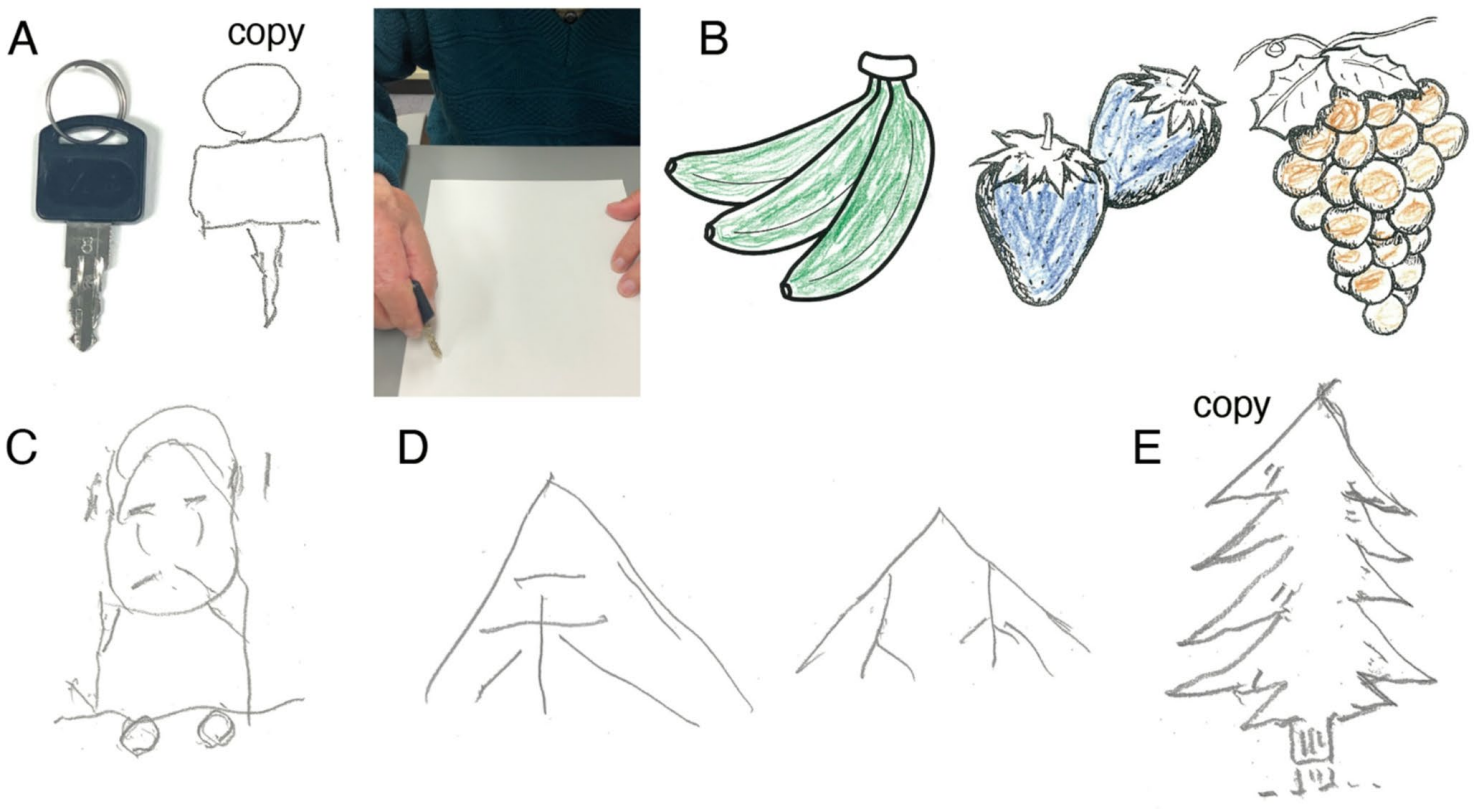
Evaluation of nonverbal object knowledge. **A**: The patient could accurately copy the key when asked to draw it; however, the patient continued to use the key as a pencil when asked to demonstrate its use. **B**: The patient demonstrated no familiarity with any of the drawings, appeared uncertain whether they were food, and colored them green, blue, and brown, respectively. **C**: The patient’s visual imagery was notably impaired, as demonstrated by the distorted and atypical drawing of a person from memory. **D**: In the delayed copy task, the drawing of a tree was markedly distorted, failing to capture its defining features (left). Although the patient could accurately copy a tree (**E**), his subsequent attempt to redraw the tree from memory immediately after was unsuccessful (**D**, right)

The patient’s impairments extended beyond language to deficits in object knowledge. He misidentified a real key as a pencil and mimicked writing with it. Although he accurately copied the key, he again used it as a pencil when asked to demonstrate its use (Fig. 2A). Semantic memory was impaired in a nonverbal pictorial semantic association task like the Pyramid and Palm Tree Test, while visual memory, assessed with the Rey-Osterrieth Complex Figure Test, was relatively preserved. When shown line drawings of bananas, strawberries, and grapes, and asked to color them appropriately, he showed no familiarity, appeared unsure if they were food, and colored them green, blue, and brown (Fig. 2B), despite intact color matching and hue discrimination. On a color-line drawing matching task (such as selecting a yellow lemon from options including red, blue, and yellow lemons), his performance was impaired (10/15), implying a deficit in conceptual knowledge. Visual imagery was notably impaired (Fig. 2C–E); in delayed copy-drawing tasks, he failed to reproduce key features of a tree seen moments earlier, despite relatively preserved constructional abilities.

These findings were consistent with SD, characterized by impaired word comprehension and semantic memory. However, given the rapid symptom progression, a repeat MRI was performed. It revealed a new subcortical hemorrhage in the left posterior inferior temporal region not seen 8 months earlier (Fig. 1B), confirmed by SWI (Fig. 1C), which also showed multiple microbleeds in the left temporal, occipital, and parietal cortices, as well as superficial siderosis (Fig. 1D). He was diagnosed with probable CAA according to the modified Boston Criteria [4]. Given the temporal correspondence between the hemorrhage and symptom deterioration, the semantic deficits were attributed to CAA rather than SD. Cerebrospinal fluid biomarkers supported coexisting AD pathology (Aβ_1−42_/Aβ_1−40_ ratio: 0.050; cut-off < 0.067). During the 20-month follow-up after the initial visit, his semantic and episodic memory further declined. Assessments at 18–20 months showed deterioration in word comprehension and the emergence of neologistic jargon. Follow-up MRI revealed progressive bilateral temporal atrophy (Fig. 1E), and increased microbleeds.

This case describes a patient with progressive semantic memory impairment resembling SD, in whom a subcortical hemorrhage in the left posterior inferior temporal region due to CAA appeared to trigger a rapid clinical decline. The combination of two-way anomia, impaired word familiarity, and deficits in object knowledge was consistent with SD. However, while the patient had a preexisting history of mild anomia possibly related to underlying neurodegenerative pathology, the emergence of marked word-comprehension deficits, reading impairment, and broader semantic dysfunction coincided with the hemorrhagic event identified on imaging, which occurred approximately 6 months prior to presentation. This temporal correspondence suggests that the hemorrhage, rather than frontotemporal degeneration, was the primary contributor to the acute worsening. Over the subsequent 20 months, although no additional large hemorrhages were observed, the progressive accumulation of microbleeds in the temporo-parieto-occipital cortices—reflecting the gradual progression of CAA—as well as advancing temporal lobe atrophy, possibly associated with AD, further contributed to the decline in language and semantic memory function.

Multiple factors may underlie the SD-like language presentation in this case. Although CAA has rarely been linked to SD-like symptoms, a possible mechanism is CAA-related hemorrhage involving the left posterior inferior temporal region—a key area for phonological-semantic mapping within the Hickok-Poeppel dual-stream model. Damage to this area can impair word comprehension, with further contribution from the involvement of the adjacent inferior longitudinal fasciculus. Additionally, the patient’s history of anomia prior to the hemorrhage suggests possible microbleeds in the left temporo-parietal cortex or an underlying AD-related process.

Regarding nonverbal semantic memory, damage to the left posterior inferior temporal region, a critical component of the ventral visual pathway for object recognition, may lead to associative visual or multimodal agnosia. According to the Patterson and Lambon Ralph’s hub-and-spoke model, this region serves as a crucial visual processing “spoke,” and its impairment can considerably disrupt semantic activation via visual input. Considering the significant roles that linguistic and visual information play in object semantics, the combination of sensory aphasia and associative agnosia possibly precipitates substantial semantic memory impairment in this case.

This case underscores the requirement for clinicians to consider CAA as a differential diagnosis of SD. CAA can cause subcortical hemorrhages and microbleeds, predominantly in the posterior temporal and occipital lobes, potentially disrupting the flow of information between language, visual, and anterior temporal regions essential for semantic processing. Its progressive nature, often accompanied by accumulating microvascular damage, may lead to gradual cognitive decline that mimics neurodegenerative disorders. Coexisting AD pathology, frequently seen in CAA, may further impair temporal lobe function. Thus, semantic memory impairment in CAA may result from both slowly progressive neurodegenerative changes and possible acute effects of hemorrhagic events. Because early MRI may miss subtle vascular changes, rapid clinical deterioration should prompt follow-up imaging. SWI is especially valuable for detecting microbleeds not visible on conventional MRI [5] and is critical when vascular contributions to cognitive impairment are suspected.

## Electronic supplementary material

Below is the link to the electronic supplementary material.

Supplementary Material 1
