# Supplementary material for: Left inferior temporal hemorrhage due to cerebral amyloid angiopathy mimicking semantic dementia
Source: Neurol Sci. 2025 Jul 11;46(10):5523–7. doi: 10.1007/s10072-025-08358-6 (PMC12488749; doi:10.1007/s10072-025-08358-6)
Supplement: Supplementary file 1 — Supplementary Material 1 [file 10072_2025_8358_MOESM1_ESM.docx]

**Supplementary Table 1.** **Results of neuropsychological tests**

| Tests |  | Initial evaluation | Follow-up evaluation  (18–20 month) | Normative data  mean (SD) |
| --- | --- | --- | --- | --- |
| ***Behavior*** |  |  |  |  |
| NPI (severity×frequency, /144) | | 5 | 27 |  |
| ***Screening*** |  |  |  |  |
| MMSE (/30) |  | 10 | 3 |  |
| Visual object naming (/7) | | 2 | 1 |  |
| Tactile object naming (/7) | | 2 | 1 |  |
| Auditory word-to-object matching (/7) | | 5 | 2 |  |
| ***Language*** |  |  |  |  |
| WAB | Aphasia Quotient (/100) | 62.3 | 47.6 | 97.7 (3.0) |
|  | Spontaneous speech (/20) | 13 | 11 | 19.7 (0.6) |
|  | Auditory comprehension (/10) | 7.55 | 4.3 | 9.8 (0.1) |
|  | Repetition (/10) | 7.5 | 8 | 9.9 (0.3) |
|  | Object naming (/60) | 16 | 3 | 59.2 (2.4) |
|  | Animal fluency (/20) | 5 | 0 | 16.2 (3.7) |
|  | Sentence completion (/10) | 4 | 0 | 9.6 (1.1) |
|  | Responsive speech (/10) | 6 | 2 | 10.0 (0.0) |
|  | Reading (/10) | 5.8 | NE | 9.5 (0.8) |
|  | Writing (/10) | 5.65 | NE | 9.6 (1.0) |
|  | Kanji word dictation (/6) | 0 | NE | 5.3 (1.5) |
|  | Kana word dictation (/6) | 3 | NE | 5.7 (1.0) |
|  | Praxis, left (/10) | 9.5 | NE | 10.0 (0.1) |
|  | Praxis, right (/10) | 9.5 | NE | 10.0 (0.1) |
| TLPA | Naming, high-familiarity words (/100) | 28 | 6 | 98.9 (2.6) |
|  | Naming, low-familiarity words (/100) | 2 | NE | 94.4 (6.4) |
|  | Auditory comprehension, high-familiarity words (/100) | 67 | 24 | 99.8 (0.8) |
|  | Auditory comprehension, low-familiarity words (/100) | 39 | NE | 99.6 (1.1) |
|  | Phonological lexical decision (/40) | 34 | NE | 39.7 (0.6) |
| 100 single-character  reading test | Kanji word reading (/100) | 60 | NE | 99.6 (1.2) |
|  | Kana word reading (/100) | 53 | NE | 99.6 (0.5) |
| Gogi aphasia task [5] | Irregular Kanji word reading (/5) | 0 | NE | 5.0 (0.0) |
|  | Proverb completion task (/6) | 2 | 1 |  |
| ***Memory*** |  |  |  |  |
| Digit span | Forward, Backward | 5, 3 | NE |  |
| Spatial span | Forward, Backward | 5, 5 | NE |  |
| ROCFT | Copy (/36) | 25 (10%ile) | NE |  |
|  | Delayed recall (/36) | 10.5 (66%ile) | NE |  |
|  | Recognition (/24) | 19 (31%ile) | NE |  |
| ***Semantic memory*** |  |  |  |  |
| Pictorial semantic association task (/16) | | 12 | 2 |  |
| Coloring task with fruit line drawings (/4) | | 0 | NE |  |
| Color-line drawing matching task (/15) | | 10 | NE |  |
| ***Visual*** |  |  |  |  |
| VPTA | Color matching (/8) | 8 | NE |  |
|  | Hue discrimination (/12) | 11 | NE |  |
|  | Coloring (/3) | 0 | NE |  |

MMSE, Mini-Mental State Examination; NE, Not Examined; NPI, Neuropsychiatric Inventory; ROCFT, Rey-Osterrieth Complex Figure Test; TLPA, Test of Lexical Processing in Aphasia; VPTA, Visual Perception Test for Agnosia; WAB, Japanese version of the Western Aphasia Battery.
